# Combined glucocorticoid and orbital radiation therapy – literature review and clinical experience

**DOI:** 10.1186/1756-6614-6-S2-A4

**Published:** 2013-04-05

**Authors:** Agata Bałdys-Waligórska

**Affiliations:** 1 Department of Endocrinology, Jagiellonian University College of Medicine, Cracow, Poland

## 

The aim of immunosuppressive treatment of Grave’s orbitopathy (GO) is to limit acute inflammation and congestion of orbital tissues. Application of intra-venous glucocorticoid (GCS) pulses is presently the treatment of choice in active (CAS≥3/7), moderate-to-severe, and severe GO. Randomised trials (RTC) have proved this treatment to be more efficient than oral glucocorticoid therapy. The response rate of this regimen is about 80%. However, exact schedules of GCS treatment have not yet been uniquely established and depend on the experience acquired in different centres.

According to the EUGOGO (2008) Consensus, to avoid acute liver damage, the total GCS dose should not exceed 8.0g in a single therapy cycle. Typically, in current schedules, the applied CGS dose per pulse per week is limited and the duration of therapy extended to 12 weeks to deliver a total methylprednisolone dose of 4.5g. While this may not always be the optimum dose, according to the EUGUGU (2012) randomised trial of the efficacy and safety of three different cumulative doses of intravenous methylprednisolone (2.5; 5.0 or 7.5 g) for moderate to severe and active GO, the dose of 7.5g was found to be most effective. It appears therefore that intravenous methylprednisolone treatment should be individualised depending on the severity of GO (NOSPECS criteria) and inflammation activity (CAS).

Orbital radiotherapy (RT) is less efficient in GO than in GCS but, as based on rather scarce RCT reports, oral glucocorticoid therapy combined with orbital radiotherapy (20 Gy) appears to give better results than GCS alone (efficacy of about 70-80%). Again, no single schedule of combined GCS and RT has been established since no randomised trials with intravenous glucocorticoid have been conducted.

According to our experience of over a decade, GCS pulse treatment followed by orbital irradiation improves the treatment outcome, especially in patients with eye muscle involvement, and reduces the frequency of recurrence.

Restoring permanent euthyroidism is very important in GO therapy. Radioiodine therapy should be considered in patients with poorly controlled hyperthyroidism. Oral GCS should be given to patients with risk factors or active GO for radioiodine use, however no randomised clinical trials have been performed to ascertain the optimum GCS dose. According to our experience, ablative 131-I doses should be applied to efficiently control thyroid function.

Radioiodine therapy can also take place while GCS pulses are delivered, with RT supplied after completion of methylprednisolone treatment. Randomised prospective trials are also necessary to assess the efficacy of these treatment schedules.

In our investigations, we have found that the group of patients who, following 131-I therapy, have reported to our Department with severe GO, demonstrated significantly higher levels of TSH and TRAb, than patients treated with anti-thyroid drugs. It is for this reason that to avoid hypothyroidism, early control of Grave’s disease patients treated with 131-I is essential.

According to the Amsterdam Declaration, detailed knowledge of the course of disease, avoiding recurrence of hyperthyroidism, and prompt qualification for well-selected treatment by an experienced endocrinologist- ophthalmologist team will improve the efficacy of GO treatment and protect the patient against severe orbitopathy.

